# Molecular Survey on Porcine Parvoviruses (PPV1-7) and Their Association with Major Pathogens in Reproductive Failure Outbreaks in Northern Italy

**DOI:** 10.3390/v16010157

**Published:** 2024-01-21

**Authors:** Giulia Faustini, Claudia Maria Tucciarone, Giovanni Franzo, Anna Donneschi, Maria Beatrice Boniotti, Giovanni Loris Alborali, Michele Drigo

**Affiliations:** 1 Department of Animal Medicine, Production and Health (MAPS), University of Padua, Viale dell’Università 16, 35020 Legnaro, Italy; giulia.faustini.1@phd.unipd.it (G.F.); giovanni.franzo@unipd.it (G.F.); michele.drigo@unipd.it (M.D.); 2 Istituto Zooprofilattico Sperimentale della Lombardia e dell’Emilia Romagna (IZSLER) “B. Ubertini”, Via Bianchi 9, 25124 Brescia, Italy; anna.donneschi@izsler.it (A.D.); mariabeatrice.boniotti@izsler.it (M.B.B.); giovanni.alborali@izsler.it (G.L.A.)

**Keywords:** porcine parvovirus, PPV1–7, PCV-2, PCV-3, PRRSV, reproductive failure, abortion, Italy

## Abstract

Successful reproductive performance is key to farm competitiveness in the global marketplace. Porcine parvovirus 1 (PPV1) has been identified as a major cause of reproductive failure, and since 2001 new species of porcine parvoviruses, namely PPV2–7, have been identified, although their role is not yet fully understood yet. The present study aimed to investigate PPVs’ presence in reproductive failure outbreaks occurring in 124 farms of northern Italy. Fetuses were collected from 338 sows between 2019 and 2021 and tested for PPVs by real-time PCR-based assays and for other viruses responsible for reproductive disease. At least one PPV species was detected in 59.7% (74/124) of the tested farms. In order, PPV1, PPV5, PPV6, PPV7 and PPV4 were the most frequently detected species, whereas fewer detections were registered for PPV2 and PPV3. Overall, the new PPV2–7 species were detected in 26.6% (90/338) of the cases, both alone or in co-infections: PCV-2 (7.1%, 24/338), PCV-3 (8.2%, 28/338), and PRRSV-1 (6.2%, 21/338) were frequently identified in association with PPVs. Single PPVs detections or co-infections with other agents commonly responsible for reproductive failure should encourage future studies investigating their biological, clinical, and epidemiological role, for a better preparedness for potential emerging challenges in intensive pig production.

## 1. Introduction

Porcine parvoviruses (PPVs) are both an old and a new challenge for the swine production sector. PPVs share many features, such as a single-stranded linear DNA genome about 5 kb long, and a non-enveloped icosahedral structure [1]. They are classified in the *Parvoviridae* family, and most of them are grouped within the *Parvovirinae* subfamily, except for the porcine parvovirus 7 (PPV7), which belongs to the *Hamaparvovirinae* subfamily [2].

Porcine parvovirus 1 (PPV1) is the common name for the most renowned species, *Protoparvovirus ungulate 1* species [2], which belongs to the *Protoparvovirus* genus [3]. Since the 1960s, PPV1 was recognized as being responsible for reproductive failure outbreaks characterized by stillbirth, mummification, embryonic death, and infertility in sows; these manifestations are commonly grouped by the acronym SMEDI. PPV1 is considered endemic, and the disease is generally controlled by vaccination; however, the virus introduction in naïve herds often causes devastating losses, effectively described as “abortion storms” [1,4,5]. Moreover, PPV1 is also known for its synergistic effect in Porcine circovirus type 2 (PCV-2) infections, affecting viremia and symptom onset [6,7,8,9]. In contrast to the broad literature examining the synergy between PPV1 and PCV-2, studies specifically investigating PPV1 interactions with Porcine circovirus type 3 (PCV-3), or Betaarterivirus suid 1 and 2 commonly known as Porcine Respiratory and Reproductive Syndrome Virus 1 and 2 (PRRSV-1; PRRSV-2) are scarce, to our knowledge.

Although PPV1 was initially thought to be a stable virus, variants emerged over the years, raising questions about the cross-protection provided by available vaccines. A particular strain, named 27a, was identified in Germany [10] and spread throughout Europe in a few years [11], showing an increased fitness due to mutations and immune escape [12].

Recently developed diagnostic techniques, such as deep sequencing, and their widespread application allowed the identification of new PPV species, namely PPV2–8, although their pathogenic role is variable and often still controversial. The first identified species was PPV2 in Myanmar in 2001 [13], and has been classified as *Tetraparvovirus ungulate 3*, genus *Tetraparvovirus*, which includes also the species *Tetraparvovirus ungulate 2* (PPV3) identified in 2008 [2,14]. PPV4 belongs to the species *Copiparvovirus ungulate 2*, genus *Copiparvovirus*, and was firstly identified in 2005 in the US [15]. PPV5, detected in 2013, is still tentatively assigned to the genus *Copiparvovirus*, due to its high similarity to PPV4 [16]. PPV6, *Copiparvovirus ungulate 4* species is also part of this genus, and it was identified in China in 2014 [17]. When PPV7 was discovered in 2016, the new *Chaphamaparvovirus ungulate 1* species was proposed, due to its high divergence from previous parvovirus species, and it was placed into the *Chaphamaparvovirus* genus. One more species (PPV8) was recently identified in 2022 and was tentatively assigned to the same genus of the original PPV1 (*Protoparvovirus*), but taxonomic classification has not been defined yet [18].

Recent literature describes new PPV2–7 circulation not only as worldwide, but it also backdates their first identification. Except for the most recent PPV7 and PPV8, all the new PPV species were detected in archive samples from the late 1990s [19,20,21], and phylodynamic estimates backdate their origin at least to 1920 (PPV2), 1930 (PPV3), and 1980 (PPV4) [22]. Moreover, their circulation is not limited to domestic pigs, as shown by their identification in wild boars as well [23,24].

Unlike PPV1, the knowledge of the pathogenicity of the new PPV2–7 is still limited and unclear. Few studies have described the presence of these new parvovirus species in pig herds, and their role in different categories of animals remains to be assessed [25,26,27,28,29,30]. PPV2–7 have been found in both healthy [28] and diseased animals (i.e., with respiratory or reproductive problems), apparently at higher frequencies in older animals, in different matrices and in coinfection with various other pathogens (especially PCV-2, PCV-3, and PPRSV), which complicates the assessment of a causal infection–disease relationship [27,29,30,31,32,33].

When cases of reproductive failure occur, the differential diagnosis includes viruses, such as PCV-2, PCV-3, and PRRSV, and bacteria associated with abortion (e.g., *Escherichia coli*, *Streptococcus* spp., *Staphylococcus* spp., *Leptospira* spp., and *Chlamydia* spp.) [4]. However, the detection of some of the new parvovirus species in aborted fetuses [25] raises questions about the completeness of routinely used diagnostic panels, which could be updated to include new suspected pathogens.

Northern Italy features a high density of pig farms, which are tightly interconnected. The comprehensive monitoring of circulating pathogens is of major interest to ensure their control, especially in the presence of disease. The preliminary evidence of the new parvovirus presence in Italy concerns wild boar and healthy pigs that are part of a controlled selection program [19,34], while studies focusing on the commercial sector are still lacking. This survey aimed to detect PPV1 and PPV2–7 species in outbreaks of reproductive failure from farms in Northern Italy and evaluates their association with other common reproductive pathogens.

## 2. Materials and Methods

### 2.1. Sampling

Samples were selected from those delivered to the Istituto Zooprofilattico Sperimentale di Lombardia ed Emilia Romagna (IZSLER, Brescia, Italy) during the period of 2019–2021, originating from farms experiencing relevant episodes of abortion, stillbirth and mummification, which are displays of reproductive failure. Swine farms were mainly located in Northern Italy, the area with the highest pork production.

Samples consisted of aborted fetuses and were identified with an individual code based on farm origin, submission, and sow. From each fetus, the target organs (liver, lungs, and heart) were collected and homogenized in pools, one for each sow. When the identification and separation of organs was not possible, fetuses were homogenized in their entirety.

### 2.2. Molecular Analysis

Aliquots of the homogenized samples were transferred to the different laboratories of IZSLER (Bacteriology laboratory and Molecular biology laboratory) in order to follow a standardized diagnostic protocol for detecting the pathogens most commonly involved in reproductive failure and circulating on the Italian territory (PCV-2, PCV-3, PRRSV-1, PRRSV-2, *Escherichia coli*, *Streptococcus suis*, *Streptococcus pyogenes*, *Staphylococcus aureus*, *Leptospira interrogans*, and *Chlamydia* spp.), herein collectively referred to as “conventional pathogens”. In particular, real-time PCRs (qPCR) were used for the detection of viral agents ([35,36], virotype PRRSV 2.0 RT-PCR Kit, INDICAL BIOSCIENCE GmbH, Leipzig, Germany), *Leptospira interrogans*, and *Chlamydia* spp., while other bacterial agents were identified by culture methods [37,38,39,40,41].

Farm metadata and diagnostic results were recorded on an Excel© (Microsoft, Albuquerque, NM, USA) file.

A second aliquot for each sample was transferred to the Infectious Disease laboratory of the Department of Animal Medicine, Production and Health (MAPS) of the University of Padua, for PPV1–7 screening. Samples were shipped and stored at −20 °C until processing.

Nucleic acids were extracted using the Viral DNA/RNA kit (A&A Biotechnology, Gdansk, Poland), following the manufacturer’s instructions. Molecular analyses were performed using QuantiFast Pathogen PCR+IC Kit (QIAGEN, Hilden, Germany) on LightCycler^®^ 96 Instrument (Roche, Basel, Switzerland), and an aliquot of internal control provided within QuantiFast Pathogen PCR+IC Kit was added during the nucleic acid extraction phase.

For screening purposes, four duplex real-time PCRs were validated based on previously published methods by Miłek et al. (2019) and Palinski et al. (2016), for the simultaneous detection of PPV1 and PPV2, PPV3 and PPV6, PPV4 and PPV5 [27], PPV7 and the internal control provided by the kit [42].

Molecular assays were validated on serial dilutions of 10^8^ copies/µL of a titrated synthetic plasmid produced by Genscript Biotech (Leiden, the Netherlands), containing the sequences targeted by the different assays (PPV1 Acc. Num. KF913351.1; PPV2 Acc. Num. GU938299; PPV3 Acc. Num. JF738367; PPV4 Acc. Num. GQ387500; PPV5 Acc. Num. JX896321; PPV6 Acc. Num. KR709268; PPV7 Acc. Num. KU563733). Samples were considered positive when the Ct was lower than 39.

### 2.3. Statistical Analysis

qPCR results were reported as binary data (e.g., positive/negative) for each pathogen tested. Absolute and relative frequencies were calculated in Microsoft Excel© (Microsoft, Albuquerque, NM, USA) and summarized in tables. For each PPV species, the association with all other conventional pathogens was tested at the individual level by a univariate analysis (Chi-square and Fisher’s exact test, when appropriate) [43,44]. The difference between years was tested for each pathogen at the farm (i.e., a farm was considered positive if at least one sample tested positive during the considered year) and individual level using a Generalized Linear Model (GLM) that included the year as a factor, and Tukey’s test was performed for multiple comparisons between years [45,46]. To explore whether the location of sampling could potentially impact pathogen frequency throughout the years, Fisher’s exact test was applied to evaluate the relationship between the variables “Year” and “Location” for the pathogen of interest. Statistical analyses were performed using R 4.3.1.

## 3. Results

### 3.1. Sample and Farm Distribution

A total of 1064 fetuses were collected from 124 farms and analyzed in 338 pooled samples; each pool was from a different sow. The number of tested sows was 74 from 44 farms in 2019, 87 from 52 farms in 2020, and 177 from 57 farms in 2021. Three farms (2.4%, 3/124) were sampled once every year. Seventeen farms were sampled in both 2019 and 2020, five in both 2020 and 2021, and one in 2019 and 2021.

**Figure 1:**
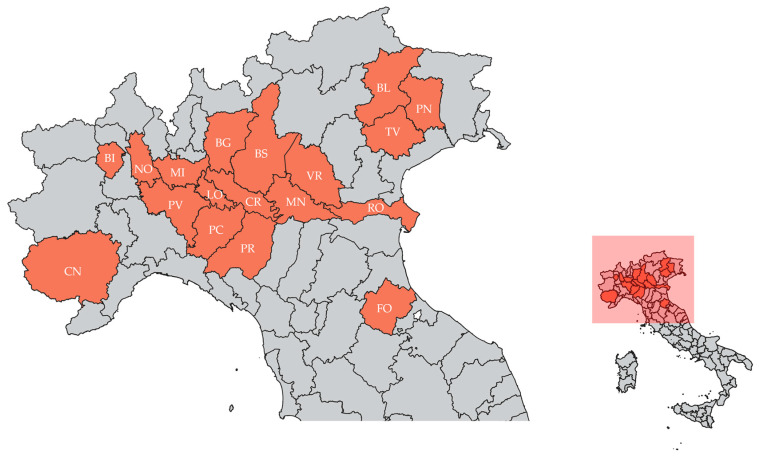
Provinces where the farms included in the study were located (red): Belluno (BL), Bergamo (BG), Biella (BI), Brescia (BS), Cremona (CR), Cuneo (CN), Forlì (FO), Lodi (LO), Mantova (MN),Milano (MI), Novara (NO), Parma (PR), Pavia (PV), Piacenza (PC), Pordenone (PN), Rovigo (RO), Treviso (TV), and Verona (VR).The list of PPV species found in each province is reported in Table S1. The map was drawn using QGIS 3.28.11 Firenze LTR.

The farms were located in 18 provinces of Northern Italy: Brescia (BS, *n* = 47), Cremona (CR, *n* = 17), Piacenza (PC, *n* = 12), Mantova (MN, *n* = 10), Bergamo (BG, *n* = 9), Cuneo (CN, *n* = 6), Milano (MI, *n* = 5), Verona (VR, *n* = 3), Biella (BI, *n* = 2), Belluno (BL, *n* = 2), Lodi (LO, *n* = 2), Pavia (PV, *n* = 2), Treviso (TV, *n* = 2), Forlì (FO, *n* = 1), Novara (NO, *n* = 1), Parma (PR, *n* = 1), Pordenone (PN, *n* = 1), and Rovigo (RO, *n* = 1) (Figure 1).

### 3.2. Trend of PPV Detection at Farm Level

**Figure 2:**
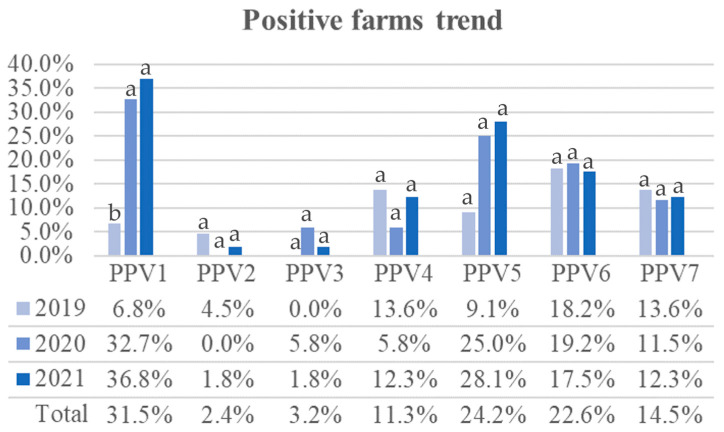
PPV-positive trends in 124 Italian farms: relative frequencies of PPV-positive farms among those analyzed in 2019 (*n* = 44), 2020 (*n* = 52), and 2021 (*n* = 57). Farms sampled in multiple years were counted only once. Significant difference between years was found only for PPV1 between 2019 and both 2020 and 2021. The letters refer to the comparison between years within each PPV species. Absolute frequencies are available in Supplementary Materials (Table S2).

All PPVs were identified in at least one farm during the period of the study (Figure 2 and Table S2), with the following Ct intervals for various detections: 8.96–38.99 for PPV1, 32.69–34.51 for PPV2, 31.94–38.59 for PPV3, 35.57–38.96 for PPV4, 31.57–38.94 for PPV5, 28.01–38.89 for PPV6, and 32.86–37.6 for PPV7.

Positive farms for at least one PPV species accounted for 43.2% (19/44) in 2019, 61.5% (32/52) in 2020, 61.4% (35/57) in 2021, and 59.7% (74/124) over the whole period, while those positive for more than one species were 13.6% (6/44), 25.0% (13/52), 31.6% (18/57), and 28.2% (35/124), respectively. The percentages of positive farms for at least one of the new PPV2–7 were 38.6% (17/44) in 2019, 44.2% (23/52) in 2020, 47.5% (27/57) in 2021, and 46.0% (57/124) over the whole period. Out of the 18 investigated provinces, 13 were positive for at least one PPV species; in Novara, Parma, Pavia, Rovigo, and Treviso, no PPVs were identified (Table S1).

PPV species persistence over the years was not observed in most of the 25 farms with a few exceptions: one farm was positive for PPV4 in 2019 and 2020, whereas three farms were positive for PPV5, one for PPV6, and one for PPV7 in 2020 and 2021.

PPV1 was the most frequently detected species, followed by PPV5, PPV6, PPV7, PPV4, PPV3, and PPV2, in decreasing order (Figure 2). In 2019, farms positive to PPV1 were significantly fewer than in 2020 (*p*-value = 0.012) and 2021 (*p*-value = 0.0043), while no significant differences among years were found for other PPV species (Table S2). The results of the Fisher’s exact test indicated a lack of statistical significance in the association between “Year” and “Location” (*p*-value = 0.8098) when considering farms and PPV1 count. Consequently, there is insufficient evidence to suggest that the sampling location played a role in the variations of PPV1 frequency.

### 3.3. Trend of Conventional Reproductive Pathogen Detection at Farm Level

All conventional pathogens (Table 1 and Table S3) were identified in at least one farm during the study period. Specifically, in 2019, 2020, and 2021, and in the overall period, positive farms for at least one conventional pathogen were 61.4% (27/44), 67.3% (35/52), 71.9% (41/57), and 69.4% (86/124), respectively, whereas those positive for more than one of them were 22.7% (10/44), 23.1% (12/52), 35.1% (20/57), and 34.7% (43/124).

Negative farms for conventional pathogens accounted for 30.6% (38/124), of which 16.9% (21/124) were also negative for all PPVs.

In the three-year-period, PCV-2 and PCV-3 were the most common findings (30.6% of the farms) (Table 1), followed by PRRSV-1 (25.0%), *E. coli* (13.7%), *S. suis* (12.9%), *Chlamydia* spp. (6.5%), and *Leptospira* spp (4.8%). PRRSV-2 was not detected. Lastly, two farms were also positive for *S. aureus* (0.8%) and *S. pyogenes* (0.8%), respectively. In 2021, PCV-3 positive farms were significantly more than in 2019 (*p*-value = 0.0212) and 2020 (*p*-value < 0.001), while no significant difference among years was found for other pathogens (Table S3). There is no significant association between “Year” and “Location” (*p*-value = 0.2128) when considering the sampled farms and PCV-3 count.

### 3.4. Frequency of PPVs and Conventional Pathogens at Individual Level over 2019–2021 Period

Over the three years, positive sows for at least one PPV species were 41.7% (141/338), whereas those positive for at least one of the new PPV2–7 were 26.6% (90/338). Positive sows for at least one conventional pathogen were 56.2% (190/338) (Table 2).

None of the tested infective agents (PPVs and conventional) were detected in 88/338 (26.0%) sows.

Viral agents were found in most cases: PPV1 was the most frequent (19.8%; 67/338), followed by PCV-3 (18.6%; 63/338), PCV-2 (17.2%; 58/338), PRRSV-1 (17.2%; 58/338), and other PPV2–7s (26.6%, 90/338) (Table 2).

Specifically, except for PPV2 and PPV3, all PPVs were also detected as single infections (Table 2). Similar to the farm level, a significant difference among years was found only for PPV1, with a significantly lower number of PPV1 cases in 2019 than in both 2020 (*p*-value = 0.0122) and 2021 (*p*-value = 0.0014) (Table S4). The Fisher’s exact test did not yield a statistically significant association between “Year” and “Location” (*p*-value= 0.1615) when considering sampled sows and PPV1 frequency; thus, there is no clear evidence that the location significantly influenced the differences in PPV1 frequency. For conventional pathogens, no statistical difference among years was found, except for PCV-3, which was detected with a higher frequency in 2021 than in 2020 (*p*-value = 0.0043). However, also for PCV-3, no significant association was demonstrated between “Year” and “Location” (*p*-value = 0.9119) when considering sampled sows and PCV-3 count.

PCV-3 (8.3%, 28/338), PCV-2 (7.1%, 24/338), and PRRSV-1 (6.2%, 21/338) was most frequently detected in coinfection with PPV species: in particular, PCV-3 with PPV1, PPV5 and PPV6; PCV-2 with PPV5 and PPV1; PRRSV-1 with PPV1 and PPV6 (Table S4). However, the only statistically significant association was between PPV1 and PRRSV-1 (*p*-value = 0.0467). In particular, PPV1 was observed more frequently in the absence of PRRSV-1.

Bacterial agents were found less frequently: *E. coli*—6.5% (22/338), *S. suis*—5.6% (19/338), *Chlamydia* spp.—3.6% (12/338), *Leptospira* spp.—1.8% (6/338), *S. pyogenes* and *S. aureus*—0.3% (1/338) (Table 2 and Table S4). No significant association was found between bacterial agents and any PPV species.

## 4. Discussion

The magnitude of PPV distribution in Italian farms is still uncertain, and the suspected pathogenic role of some of the new PPV species emphasizes the need to understand their dissemination and circulation. The present study is the first survey investigating PPV species presence in Italy in commercial pigs. PPV1 has been commonly identified both molecularly and serologically in domestic and wild populations [47,48,49], whereas other species have been reported more sporadically. For example, PPV3 was detected in a dead wild boar in Southern Italy for the first time in 2016 [34]. The other PPVs, except for PPV7 and PPV8, were identified by retrospective metagenomic studies using samples from breeding pigs reared in the period 1996–2007 [19]; however, no hypothesis about their pathogenic relevance was inferred because all animals were considered healthy at the time of sampling [19,34].

PPV1 is an endemic pathogen and is thus routinely monitored [50]. The occurrence of PPV1-related clinical signs is controlled by means of vaccination, which prevents disease but often fails in blocking infection and viral transmission. Therefore, a certain degree of PPV1 circulation can reasonably be expected in Italy.

Nevertheless, a report on the diagnostic activity performed in the same geographic area in the period 2011–2013 described 3.6% of PPV1-positive cases [37], which remarkably differed from the 19.8% herein detected for the period of 2019–2021. Other than a considerable variation in the viral epidemiology, a substantial difference in the sensitivity of the diagnostic assays used in the two studies (i.e., quantitative PCR instead of viral isolation) has to be considered as a possible underlying reason for this discrepancy. However, changes in the epidemiological scenario are likely, and an actual increase in PPV1 frequency should not be overlooked, especially when considering its synergistic effect with other pathogens in determining reproductive failures. Moreover, the wide viral circulation, in spite of the largely adopted PPV1 vaccination, could motivate a reassessment of the prophylactic strategies in light of the potential emergence of new PPV1 strains [51]. This consideration aligns with the suggested role of vaccines as drivers of evolution [11,52]. Dedicated studies characterizing PPV1 circulating strains are needed to further unravel the molecular aspects of its epidemiology in Northern Italy.

The significant increase in PPV1 cases witnessed in 2020–2021 may have been also influenced by local management factors, impairing the effectiveness of general preventive and control measures. Therefore, any extension of these findings to different swine populations needs to be confirmed by dedicated studies. However, the present scenario is only partially supported by other pathogens, since a similar increasing trend was evident only for PCV-3. Additionally, the lack of a clear influence of the sampling location on the differences in PPV1 and PCV-3 frequency between the years 2019, 2020, and 2021, may indicate that other factors, not captured by this analysis, could contribute to the observed patterns. Since the absence of a significant association does not necessarily imply the absence of a real-world effect, additional investigations and a broader understanding of the interplay of many factors warrant assessment, given the peculiarity of such an abrupt increase in PPV1 circulation.

PPV1 predominance was not the only relevant finding, and the identification of PPV2–7 species is worth discussing. The present study confirms the circulation of PPV2-6 species and describes for the first time the presence of PPV7 in Italy. The detection of new PPVs, either as single agents, except for PPV2 and PPV3, or in co-infection with other conventional pathogens in aborted fetuses, demands the investigation of their potential primary and/or synergistic etiological role in reproductive pathology.

In particular, this is the first identification of PPV2, PPV3 and, most frequently, PPV5 in reproductive failure outbreaks. The identification of PPV4, PPV6, and PPV7 in reproductive failure cases, as single agents and co-infections within the tested panel, is consistent with evidences from other countries, fueling hypotheses on their pathogenicity [17,25,53,54]. The different frequencies observed for PPVs could be biased by their different tissue tropism and differing circulation within various animal production categories. Indeed, available reports depict oral fluids and serum as matrices with the highest detection rates for almost all PPVs, mostly in growing pigs [27,55]. Hence, the frequencies recorded in this study may be underestimated, especially for PPV2 and PPV3. However, Csagola et al. [25] detected PPV4 in fetal tissues with higher frequency than in other matrices. This finding, together with the here-presented higher prevalence of other PPV species, may be indicative of their ability to cross the placental barrier and be vertically transmitted. However, only dedicated studies would better elucidate the PPV2–7 pathogenesis.

The data here presented showed the co-circulation of several microbial agents in farms experiencing reproductive failure outbreaks. Bacterial agents may have acted mainly as opportunistic pathogens, although *E. coli* and *Streptococcus* spp. in single infections may have been sufficient to cause abortion [4,56]. Conversely, the central role of viral agents in determining reproductive failures has been proven [57,58]. Viral agents could both systemically and locally affect reproductive organs, causing systemic dysfunctionality and damage in fetal and maternal tissues, potentially favoring bacterial growth, and leading to abortion [59]; thus, PPV2–7 might act in both ways. The currently available literature describing PPVs in the presence of PCV-2 and PCV-3 is wide, especially for the association between PPVs and PCV-2 [20,32,60,61,62,63,64]. PPV1, PPV2, and PPV7 are described as favoring PCV-2-infection [21,31], while only PPV7 appears to stimulate the PCV-3 replication, contributing as a co-factor in PCV-3-associated reproductive disorders [63]. In our survey, a partially different scenario was uncovered, where PCV-2 was more frequently retrieved in coinfection with PPV5 than with PPV1, and the co-infection rate between PCV-3 and PPV7 was considerably lower. Given this evidence, the existence of synergies among PPV2–7 and porcine circoviruses should be further investigated [65,66,67,68].

PPV species were also found in coinfection with PRRSV-1, whose role as a primary etiological agent in reproductive failure has been more deeply investigated than its synergetic action with PPV1 and new PPV2–7. Considering the immunomodulatory effect of PRRSV, a synergetic effect in triggering and exacerbating reproductive disorders can be suspected [69]. Although the lower frequency of PPV1 in the presence of a high frequency of PRRSV-1 observed in this study might suggest the opposite, other explanations may be advocated, since this apparent association is likely biased by the type of sampling. Considering that an actual protective effect of PRRSV would not be biologically reasonable, dedicated studies are needed to further substantiate the interactions of PRRSV with PPVs.

As already discussed, the results of the present study could be strongly affected by convenience/opportunistic sampling and by the choice of the investigated matrix, and it cannot be excluded that more farms could have tested positive for PPV2 and PPV3, if others and possibly more suitable matrices, such as serum, had been analyzed [20]. Similarly, different viral or viral genome persistence in the tested tissues might have affected the absolute and relative detection frequency of the considered pathogens, also because of the fetus age and/or decomposition stage. Furthermore, this survey was conducted on fetuses actively collected by the farmer/veterinarian. Early abortions may result in fetal reabsorption that could go unnoticed and be misinterpreted as infertility, and contribute to the underestimation of the actual impact of PPVs in reproductive syndromes. Additionally, this study did not investigate anatomopathological aspects, preventing conclusions on the real pathogenetic contribution of the detected pathogens, nor did it account for non-infective causes of reproductive problems [70]. Environmental factors, toxins, poor management, and other stressors could have been involved in the clinical episodes in farms negative to all tested infective agents, thus inflating the study denominators. Conversely, this study does not investigate all pathogens responsible for reproductive problems, leaving the cases of the negative sows undiagnosed. The analysis of serum, fetal fluids, and vaginal swabs from all sows showing infertility, also considering environmental and managerial contexts, would allow for a more extensive understanding of the infection relevance in episodes of reproductive failure [59]. Moreover, the analysis of samples actively submitted by veterinarians prevents prevalence estimations, thus a dedicated survey on both healthy and diseased animals, belonging to different livestock categories, would provide a broader understanding of PPVs’ epidemiology, which is crucial for countries renowned for swine farming, like Italy.

## 5. Conclusions

The present study is the most extensive survey performed so far in Italy, describing the circulation of the new parvovirus species PPV2–7, alone and in co-infection with a panel of other pathogens commonly responsible for reproductive failure. Although the identification of new parvovirus species in samples from reproductive failure outbreaks might be suggestive of a potential clinical role, the causal nexus cannot be confidently stated. Thus, further dedicated and standardized studies considering healthy and diseased animals, different matrices, and production categories are needed to verify the real impact of PPV2–7 and describe their prevalence in the productive context. Evidence of the spread of new PPV species and high PPV1 circulation highlight the importance of the monitoring of emerging and newly discovered pathogens, such as PPV8, which should be included in future surveys. Together with previous reports, the identification of PPV4, PPV5, PPV6, and PPV7 as single agents in diseased subjects should prompt their inclusion in routinely applied diagnostic protocols to monitor their occurrence in cases of reproductive failure. Moreover, the high frequency of PPV1 warrants the need for the characterization of circulating strains, in order to update the epidemiological knowledge and assess the suitability of current vaccines.

## Figures and Tables

**Table 1:** Absolute and relative frequencies of positive farms for conventional pathogens among those tested in 2019 (*n* = 44), 2020 (*n* = 52), and 2021 (*n* = 57). The total frequency refers to 124 farms, since farms sampled in multiple years were counted only once.

Year	PRRSV-1	PCV-2	PCV-3	Cultured Bacteria *	*L. interrogans*	*Chlamydia* spp.
	*n* (%)	*n* (%)	*n* (%)	*n* (%)	*n* (%)	*n* (%)
2019	7 (15.9)	13 (29.5)	8 (18.2)	11 (25.0)	0 (0.0)	3 (6.8)
2020	14 (26.9)	16 (30.8)	5 (9.6)	11 (21.2)	4 (7.7)	1 (1.9)
2021	15 (26.3)	12 (21.1)	25 (43.9)	11 (19.3)	2 (3.5)	4 (7.0)
Total	31 (25.0)	38 (30.6)	38 (30.6)	31 (25.0)	6 (4.8)	8 (6.5)

* Cultured bacteria include positivity for *E. coli*, *S. suis*, *S. pyogenes* and/or *S. aureus*.

**Table 2:** Absolute and relative frequencies of infective agents as single infections and in co-infection with other pathogens in samples. Only the column of single infections is indicative of a single agent detection. The sum of columns reporting the association with other pathogens does not equal the total number of cases, because they include single and multiple coinfections. A complete matrix with single frequencies of coinfection between conventional pathogens and each PPV is reported in Table S5.

			Association with								
Infective Agent	Total Cases	Single Infections	Grouped PPV2–7	PRRSV-1	PCV-2	PCV-3	*E. coli*	Strept. spp.*	*S. aureus*	*L.* *interrogans*	*Chlamydia* spp.
	*n* (%) ^1^	*n* (%) ^2^	*n* (%) ^2^	*n* (%) ^2^	*n* (%) ^2^	*n* (%) ^2^	*n* (%) ^2^	*n* (%) ^2^	*n* (%) ^2^	*n* (%) ^2^	*n* (%) ^2^
PPV1	67 (19.8)	33 (49.3)	16 (23.9)	6 (9.0)	7 (10.4)	17 (25.4)	4 (6.0)	4 (6.0)	1 (1.5)	2 (3.0)	1 (1.5)
PPV2	3 (0.9)	0 (0.0)	2 (66.7)	1 (33.3)	0 (0.0)	0 (0.0)	0 (0.0)	0 (0.0)	0 (0.0)	0 (0.0)	0 (0.0)
PPV3	4 (1.2)	0 (0.0)	2 (50.0)	1 (25.0)	2 (50.0)	1 (25.0)	1 (25.0)	0 (0.0)	0 (0.0)	0 (0.0)	0 (0.0)
PPV4	15 (4.4)	4 (26.7)	4 (26.7)	2 (13.3)	4 (26.7)	1 (6.7)	2 (13.3)	1 (6.7)	0 (0.0)	0 (0.0)	1 (6.7)
PPV5	38 (11.2)	11 (28.9)	11 (28.9)	5 (13.2)	8 (21.1)	7 (18.4)	1 (2.6)	3 (7.9)	0 (0.0)	2 (5.3)	0 (0.0)
PPV6	31 (9.2)	7 (22.6)	13 (41.9)	6 (19.4)	5 (16.1)	7 (22.6)	0 (0.0)	4 (12.9)	0 (0.0)	1 (3.2)	0 (0.0)
PPV7	20 (5.9)	7 (35.0)	5 (25.0)	2 (10.0)	5 (25.0)	3 (15.0)	2 (10.0)	1 (5.0)	0 (0.0)	0 (0.0)	0 (0.0)
Grouped PPV2–7	90 (26.6)	37 (41.1)		15 (16.7)	19 (21.1)	16 (17.8)	6 (6.7)	7 (7.8)	0 (0.0)	3 (3.3)	1 (1.1)
PRRSV-1	58 (17.2)	26 (44.8)	15 (25.9)		6 (10.3)	8 (13.8)	5 (8.6)	2 (3.4)	0 (0.0)	0 (0.0)	1 (1.7)
PCV-2	58 (17.2)	26 (44.8)	19 (32.8)	6 (10.3)		8 (13.8)	3 (5.2)	3 (5.2)	0 (0.0)	1 (1.7)	2 (3.4)
PCV-3	63 (18.6)	18 (28.6)	16 (25.4)	8 (12.7)	8 (12.7)		5 (7.9)	4 (6.3)	0 (0.0)	2 (3.2)	3 (4.8)
*E. coli*	22 (6.5)	6 (27.3)	6 (27.3)	5 (22.7)	3 (13.6)	5 (22.7)		3 (13.6)	0 (0.0)	1 (4.5)	1 (4.5)
*Streptococcus* spp.	20 (5.9)	4 (20.0)	7 (35.0)	2 (10.0)	3 (15.0)	4 (20.0)	3 (15.0)		0 (0.0)	2 (10.0)	1 (5.0)
*S. aureus*	1 (0.3)	0 (0.0)	0 (0.0)	0 (0.0)	0 (0.0)	0 (0.0)	0 (0.0)	0 (0.0)		0 (0.0)	0 (0.0)
*L.* *interrogans*	6 (1.8)	1 (16.7)	3 (50.0)	0 (0.0)	1 (16.7)	2 (33.3)	1 (16.7)	2 (33.3)	0 (0.0)		0 (0.0)
*Chlamydia* spp.	12 (3.6)	6 (50.0)	1 (8.3)	1 (8.3)	2 (16.7)	3 (25.0)	1 (8.3)	1 (8.3)	0 (0.0)	0 (0.0)	

^1^ = relative frequencies refer to the total number of tested sows (*n* = 338); ^2^ = relative frequencies of other column refer to total cases for that infective agent; * = *Streptococcus* spp. includes *S. suis* and *S. pyogenes*.

## Data Availability

Data is contained within the article or supplementary materials.

## Supplementary Materials

The following supporting information can be downloaded at: https://www.mdpi.com/article/10.3390/v16010157/s1, Table S1: Absolute and relative number of PPV-positive farms in each province investigated; Table S2: Absolute and relative frequencies of PPV positive farms in 2019 (*n* = 44), 2020 (*n* = 52), and 2021 (*n* = 57); Table S3: Absolute and relative frequencies of positive farms for all tested conventional pathogens in 2019 (*n* = 44), 2020 (*n* = 52), and 2021 (*n* = 57); Table S4: Absolute and relative frequency of positive sows in 2019 (*n* = 74), 2020 (*n* = 87), and 2021 (*n* = 177) for each PPV species; Table S5: Complete matrix with single frequencies of coinfection between conventional pathogens and each PPVs in tested samples.
